# Vector competence of *Culex* mosquitoes (Diptera: Culicidae) in Zika virus transmission: an integrative review

**DOI:** 10.26633/RPSP.2020.7

**Published:** 2020-02-03

**Authors:** Sandro G. Viveiros-Rosa, Eduardo G. Regis, Wilson C. Santos

**Affiliations:** 1 Programa de Pós-Graduação em Ciências Aplicadas a Produtos Para Saúde, Faculdade de Farmácia Universidade Federal Fluminense NiteróiRio de Janeiro Brasil Programa de Pós-Graduação em Ciências Aplicadas a Produtos Para Saúde, Faculdade de Farmácia, Universidade Federal Fluminense, Niterói, Rio de Janeiro, Brasil.; 2 Instituto Nacional da Propriedade Industrial Divisão de Biofármacos Rio de Janeiro Brasil Instituto Nacional da Propriedade Industrial, Divisão de Biofármacos, Rio de Janeiro, Rio de Janeiro, Brasil.

**Keywords:** Public health, Zika virus, mosquito vectors, *Culex.*, Salud pública, virus Zika, mosquitos vectores, *Culex.*, Saúde pública, Zika virus, mosquitos vetores, *Culex.*

## Abstract

**Objective.:**

To identify studies on the competence of *Culex* mosquitoes as vectors for the transmission of Zika virus (ZIKV) around the globe.

**Methods.:**

We performed an integrative review to identify relevant articles on specific experiments to determine whether *Culex* mosquitoes are vectors for ZIKV. The sources we used for our research were the Brazilian Periódicos CAPES electronic portal (MEDLINE/PubMed, ScienceDirect Journals, Nature Publishing Group, SciELO, Springer Link, and 250 other databases) and gray literature.

**Results.:**

We identified 344 studies, of which 36 were considered for this review. In 8 studies, infection in salivary glands of *Culex quinquefasciatus*, *Culex restuans, Culex tarsalis*, and *Culex coronator* was detected. *Cx. quinquefasciatus* was the most studied among those confirmed as potential ZIKV vectors, and only strains of Asian lineages (THA/2014/SV0127-14; SZ01 (2016)) and American lineages (BRPE243 (2015); PRVABC59 (2015)) can infect the salivary glands of *Culex* mosquitoes. The tested African strains (MR766 and DAK AR 41525) were unable to infect salivary glands.

**Conclusions.:**

There is still a lack of compelling evidence that indicates *Culex* spp. are a competent ZIKV vector, but they should remain a target for further monitoring studies, especially regarding ZIKV transmission to other species. Furthermore, studies should not be limited to studying whether their salivary glands are infected.

## RESUMEN

**Objetivo.:**

Identificar estudios sobre la competencia de los mosquitos *Culex* como vectores de la transmisión del virus del Zika en todo el mundo.

**Métodos.:**

Se realizó una revisión integradora para identificar artículos relevantes sobre experimentos específicos dirigidos a determinar si los mosquitos *Culex* son vectores del virus del Zika. Se emplearon fuentes obtenidas a partir del portal electrónico de revistas CAPES (MEDLINE/PubMed, ScienceDirect Journals, Nature Publishing Group, SciELO, Springer Link, y otras 250 bases de datos) y la literatura gris.

**Resultados.:**

Se identificaron 344 estudios, 36 de los cuales fueron considerados para esta revisión. En 8 estudios se detectó infección en las glándulas salivales de *Culex quinquefasciatus, Culex restuans, Culex tarsalis* y *Culex coronator*. *Cx. quinquefasciatus* fue la especie más estudiada entre las confirmadas como potenciales vectores del virus del Zika, y solo las cepas de linajes asiáticos (THA/2014/SV0127-14; SZ01 [2016]) y americanos (BRPE243 [2015]; PRVABC59 [2015]) pueden infectar las glándulas salivales de los mosquitos *Culex*. Las cepas africanas analizadas (MR766 y DAK AR 41525) no fueron capaces de infectar las glándulas salivales.

**Conclusiones.:**

Aunque faltan pruebas convincentes que indiquen que las especies de *Culex* spp. son un vector competente del virus del Zika, estas deben seguir monitoreándose mediante estudios adicionales, especialmente respecto de su capacidad para transmitir el virus del Zika a otras especies. Esta vigilancia no debería limitarse solamente a determinar la infección en las glándulas salivales.

## RESUMO

**Objetivo.:**

Identificar estudos sobre a competência dos mosquitos *Culex* como vetores da transmissão do vírus Zika em todo o mundo.

**Métodos.:**

Uma revisão integrativa foi realizada para identificar artigos relevantes sobre experimentos específicos para determinar se os mosquitos *Culex* são vetores do vírus Zika. As fontes utilizadas na pesquisa foram o portal eletrônico CAPES (MEDLINE/PubMed, ScienceDirect Journals, Nature Publishing Group, Sci-ELO, Springer Link, e outras 250 bases de dados) e a literatura cinza.

**Resultados.:**

Foram identificados 344 artigos, dos quais 36 foram considerados para esta revisão. Oito artigos relataram infecção nas glândulas salivares de *Culex quinquefasciatus, Culex restuans, Culex tarsalis* e *Culex coronator. Culex quinquefasciatus* foi a espécie mais estudada entre as confirmadas como vetores potenciais do vírus Zika. Apenas as linhagens asiáticas (THA / 2014 / SV0127-14; SZ01 [2016]) e americanas (BRPE243 [2015]; PRVABC59 [2015]) podem infectar as glândulas salivares dos mosquitos *Culex*. As cepas africanas analisadas (MR766 e DAK AR 41525) não foram capazes de infectar as glândulas salivares.

**Conclusões.:**

Ainda não há evidências convincentes para indicar que os mosquitos *Culex* são um vetor competente do vírus Zika. Contudo, estudos adicionais de monitoramento devem ser realizados, especialmente no que diz respeito à transmissão do vírus Zika para outras espécies de mosquitos. Além disso, os estudos não devem se limitar a estudar a infecção nas glândulas salivares.

Zika virus (ZIKV) is known to be transmitted among humans mainly through bites of *Aedes aegypti* (Linnaeus) and *Aedes albopictus* (Skuse) mosquitoes (1). The virus was initially isolated in a rhesus monkey in 1947. There was a second isolation from *Aedes africanus* (Theobald) in 1948 in an attempt to isolate yellow fever virus from mosquitoes in the Zika Forest of Uganda (2). *Aedes* mosquitoes are considered the only competent vectors for ZIKV transmission (3, 4). Transmission can occur sexually (5), through blood transfusion and saliva, and from mother to child during pregnancy, birth, and breast-feeding (6).

ZIKV is a positively enveloped RNA virus member of the *Flaviviridae* family, genus *Flavivirus* (1, 7). It was discovered in 1947 in the Zika Forest, in Uganda, and remained confined to some areas of Africa and Asia. In 2007, ZIKV emerged in the Yap Islands in the Federated States of Micronesia and also in the African country of Gabon. In addition, in 2013, the virus appeared in French Polynesia. By 2014, ZIKV had spread to other Pacific islands: New Caledonia, the Cook Islands, and Easter Island. In early 2015, the virus was identified in Brazil and then, later, throughout continental South America and Latin America (1, 7). This fast and massive spread is worrisome because there are no available drugs or vaccines for the treatment of ZIKV infection, and a possible marked, severe outcome of ZIKV infection in pregnant women is microcephaly in newborns (6).

Within the genus *Aedes*, other anthropophilic species have been considered to be vectors, including *Aedes hensilli* (Farner) and *Aedes vexans* (Meigen). In addition, other mosquito genera have been investigated as vectors, especially *Culex*. (3, 4, 8, 9). *Culex* spp. are already considered competent vectors to transmit such flaviviruses as human-like Japanese encephalitis virus (JEV), West Nile virus (WNV), and Saint Louis encephalitis (10). This fact makes them candidates for further investigations as a vector for other flaviviruses, such as ZIKV.

In a study performed in China, *Culex quinquefasciatus* (Say) was identified as a potential laboratory vector for ZIKV. In that research, mosquitoes were infected through a blood meal with a ZIKV strain (SZ01) isolated from a patient, and viral RNA was found in the salivary glands, midgut, and ovary. Importantly, *Cx. quinquefasciatus* mosquitoes were able to infect infant mice (11). In a session of the First International Workshop on Zika Virus, organized by the Oswaldo Cruz Foundation (FIOCRUZ) and held in Brazil in 2016, researchers from the Ageu Magalhães Institute (Recife, Pernambuco, Brazil) presented results of experiments with mosquitoes artificially fed with blood infected with the ZIKV. The virus was detected in the body and in salivary glands of *Cx. quinquefasciatus* 7 and 15 days after feeding, with a confirmed infection rate of 100% and 67%, respectively (12).

Currently, there are no vaccines or drugs against ZIKV (1, 4, 5), and the only precaution is to prevent mosquito bites by integrated vector control, including surveillance, biological and chemical control, source reduction, and environmental management (4, 5). For example, the development of sanitization and management of urban water collection and vector control with DDT was responsible for vector *Aedes* mosquitoes disappearing after 1950, although the species has recently reinvaded European territory (4). Furthermore, the World Health Organization (WHO) also recommends the practice of safe sex for women living in areas of high virus transmission (13).

Therefore, considering that the genus *Aedes* is the principal target among the strategies for the prevention and control of ZIKV, we reasoned that it is essential to understand whether there are other vectors capable of or even competent in transmitting ZIKV. Thus, we aimed to identify studies on the competence of the *Culex* mosquitoes as vectors for ZIKV transmission through a review of the literature on this subject.

## METHODS

We performed a literature review in July 2019 to identify relevant articles on the vector competence of *Culex* spp. mosquitoes for ZIKV. The review was based on an advanced search of the Brazilian Periódicos CAPES electronic portal (www.periodicos.capes.gov.br), which includes MEDLINE/PubMed, ScienceDirect Journals, Nature Publishing Group, SciELO, Springer Link, and 250 other databases. Additional studies were identified by searching for gray literature, using the Google Scholar search engine, and with a manual search of the bibliographical references of the relevant identified publications.

The search strategy was drawn from the descriptors (“Culex” and “vector”) AND (zik*), marking “any field” for the search field of the thesauri, in the advanced-search area of the Periódicos CAPES electronic portal. All studies from the literature search were analyzed to eliminate duplicates from the manual search strategies, specifically by comparing authors, titles, and name of the journal, along with their volume, number, and year of publication. After the duplicates were removed, the remaining studies were examined based on their titles and abstracts. At this stage, the eligibility criteria for the articles were: original studies; mentions of ZIKV and a mosquito from the genus *Culex;* establishes *Culex* spp. as a vector of ZIKV through specific results in experiments identifying the virus at their organisms; and in Portuguese, Spanish, or English. Reviews and opinion letters were discarded. The documents selected as potentially relevant went to the next step, where they were judged carefully from the reading of the full text. Only those that met all the eligibility criteria mentioned above were included in this review. The studies from each database were placed in spreadsheets in the Microsoft Excel program (version 2010), to eliminate duplicates and to create a database for references.

## RESULTS

The study designs were classified as cohort studies (10 articles), case-control studies (26 articles), and a conference abstract (1 article). One of the documents presented two different study classifications, case-control and cohort.

### Study selection

**FIGURE 1.**
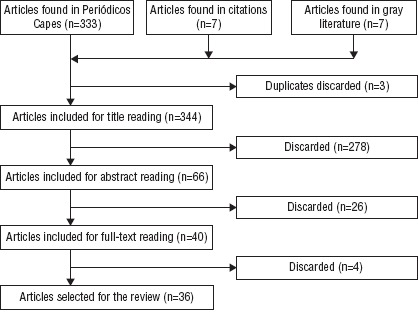
Flow diagram of the methodology used to identify studies.

***Source:*** prepared by the authors

We identified 333 studies in the bibliographic search in the Periódicos CAPES website, 7 studies through gray literature, and 7 additional studies from the manual search of the bibliographical references of the relevant publications, for a total of 347 studies. Three duplicate papers were discarded, and then 278 articles were excluded after reading the title. We incorporated 66 studies whose titles were not clear concerning inclusion criteria into the next stage, which included reading the abstracts to avoid missing any article that matched our criteria (all the articles were written in English, so no study was excluded based on language). In the end, after discarding 26 articles by reading the abstract and 4 articles by reading the full text, we included 36 studies in this review. Figure 1 is a flow diagram of the methodology steps. Table 1 summarizes the articles used in this review.

**TABLE 1.** Studies included in the review.

Author (Reference)	Study Design	Country/Year	Blood Meals Titers	Infection	S.G Infection	Observations
Guo et al. (11)	CC	China 2016	3x10^5^ PFU/ml	**+**	**+**	89% of mice infected had viral RNA of strain SZ01 in their brain at 10 DPI with 7.85 log RNA copies/ml. *Cx. quinquefasciatus* tested.
Liu et al. (37)	CC	China 2017	5.45 ± 0.38 log_10_ Copies/ml	**+**	**-**	Strain ZJ03 used. 15,94 % of *Cx. quinquefasciatus* infected.
Fu et al. (33)	CH	China 2017	MC	**+**	NT	*Cx. quinquefasciatus* collected from the field with strain GZDJ1685-2 detected.
Phumee et al. (47)	CC	Thailand 2019	1.7x10^5^ FFU/ml	**+**	**+**	Vertical transmission of ZIKV in *Ae. aegypti, Ae. albopictus and Cx. quinquefasciatus* confirmed. Strain used: Zika virus/H. sapiens-tc/THA/2014/SV0127-14.
Pompon et al. (34)	CC	Singapore 2017	10^5^ to 10^6^ PFU/ml	**+**	**-**	Strain H/PF used. *Cx. quinquefasciatus* tested.
Ho et al. (25)	CH	Singapore 2017	MC	**-**	**-**	*Aedes aegypti* infected in many pools. No *Culex* spp. infected.
Tien et al. (26)	CH	Singapore 2017	MC	**-**	**-**	*Aedes aegypti* infected in many pools. No *Culex* spp. infected.
Franca et al. (12)	CA	Brazil 2016	Not revealed	**+**	**+**	Transmission to other species not tested.
Guedes et al. (43)	CC; CH	Brazil 2017	10^6^ PFU/ml and MC	**+**	**+**	A study made with mosquito *Cx. quinquefasciatus* and strain BRPE243/2015. Collected mosquitoes with salivary glands infected. No traces of recent feeding.
Fernandes et al. (31)	CC	Brazil 2016	10^6^ PFU/ml	**+**	**-**	Strains Rio-U1 and Rio S-1 tested. 3.3% of *Cx. quinquefasciatus* infected.
Fernandes et al. (35)	CC	Brazil 2017	2.3 × 10^6^, 3.55 × 10^6^ and 1.68 × 10^7^ PFU/ml	**+**	**-**	5% of *Cx. quinquefasciatus* infected. Strains: BRPE243/2015, U1, and SPH2015.
Ayres et al. (42)	CH	Brazil 2019	MC	**+**	NT	A study made with *Cx. quinquefasciatus* collected from the field at Espírito Santo. *Ae. aegypti* also infected. Strains detected: PE243 2015 (from Pernambuco), Haiti 1225 2014, and SPH2015 (from São Paulo).
Lourenço-de-Oliveira et al. (23)	CC	Brazil 2018	10^7^ TCID_50_/ml	**-**	**-**	*Cx. quinquefasciatus* tested with strains NC-2014-5132.
Dibernardo et al. (44)	CC	Canada 2017	10^5.4^ PFU/ml	**+**	**+**	Intrathoracic inoculation of strain PRVABC59 of ZIKV at *Cx. restuans* and *Cx. tarsalis*. In 3/58 of *Cx. restuans* was detected ZIKV in the salivary glands.
Smartt et al. (46)	CC	United States 2018	3.5 log_10_ ZIKV PFUe/ml titer	**+**	**+**	A study performed with *Cx. quinquefasciatus* and strain PRVABC59. RNA analysis at saliva eluted from the filter paper at 14 (Exp.1) and 16 DPI (Exp2) to detect ZIKV titers.
Weger-Lucarelli et al. (32)	CC	United States 2016	1.6x10^7^ and 5.0x10^6^ PFU/ml	**+**	**-**	2% of *Cx. quinquefasciatus* infected. *Cx. pipiens* nor *Cx. tarsalis* not infected. Strain: PRVABC59
Hart et al. (20)	CC	United States 2017	10^4^, 10^5^, 10^6^ and 10^7^(FFU/ml)	**-**	**-**	*Cx. quinquefasciatus* tested with strains FSS13025, PRVABC59, MEX1–7, and DAK AR 41525.
Kenney et al. (38)	CC	United States 2017	4, 5.9, 6, 7.1 and 7.6 log_10_ PFU/ml	**+**	**-**	1-10% of mosquitoes infected. 15–70% of mosquitoes intrathoracically inoculated demonstrated virus-positive bodies. Mosquitoes of *Cx. quinquefasciatus* and *Cx. pipiens*. Strains: MR766, R103451 and PRVABC59.
Main et al. (40)	CC	United States 2018	4.6 log_10_ PFU/m	**+**	**-**	30% of infection at 21 DPI for *Cx. tarsalis*. Dissemination of strain PRVABC59 at 4% at 14 DPI and 5% at 21 DPI. *Cx. quinquefasciatus* not infected.
Dodson et al. (18)	CC	United States 2017	7.3 and 7.5 log_10_ PFU/ml ^10^	**-**	**-**	*Cx. quinquefasciatus* tested with strains PRVABC59 and MR766.
Dodson et al. (22)	CC	United States 2018	8.4 log_10_ PFU/ml	**-**	**-**	*Cx. tarsalis* tested with strain NR-43026.
Aliota et al. (14)	CC	United States 2016	4.74, 6.02 and 6.83 log_10_ PFU/ml	**-**	**-**	*Cx. pipiens* tested with strain PRVABC59.
Huang et al. (17)	CC	United States 2016	106.52, 106.95, 107.52 logTCID_50_/ml	**-**	**-**	*Cx. pipiens* and *Cx. quinquefasciatus* tested with strain PRVABC59.
Yee et al. (21)	CH	Puerto Rico 2017	MC	**-**	**-**	*Cx. quinquefasciatus* not infected with no ZIKV. *Aedes aegypti* infected.
Elizondo-Quiroga et al. (45)	CH	Mexico 2018	MC	**+**	**+**	Mosquitoes *Cx. quinquefasciatus, Cx. coronator* and *Cx. tarsalis*. Strains not identified. 2/5 of SG pools for *Cx. quinquefasciatus* showed CPE at 1 DPI. Pools of SG of *Cx. coronator* and *Cx. tarsalis* showed CPE at 3 and 4, respectively. *Ae. aegypti* showed the lowest MIR/1000 mosquitoes.
Guerbois et al. (16)	CH	Mexico 2016	MC	**-**	**-**	A study performed with *Cx. quinquefasciatus* collected. *Ae. aegypti* infected.
Diallo et al. (30)	CH	Senegal 2014	MC	**+**	NT	A study made with *Cx. perfucus* collected from the field. 1/7 pools infected. Strains not identified.
Amraoui et al. (28)	CC	Tunisia 2016	10^7.2^ PFU/ml	**+**	**-**	*Cx. pipiens* and *Cx. quinquefasciatus* inoculated intrathoracically with ca. 2,530 PFU. Strain NC2014-5132 used.
Boccolini et al. (29)	CC	Italy 2016	10^6.46^ PFU/ml	**+**	**-**	Strain H/PF for *Cx. pipiens*.
Foxi et al. (15)	CH	Italy 2016	MC	**-**	**-**	A study performed with *Culex* spp.
Heitmann et al. (36)	CC	Germany 2017	10^7^ PFU/ml	**+**	**-**	Strain FBGWUH2016. *Cx. molestus, Cx. pipiens* and *Cx. torrentium* infected but with no dissemination nor transmission detectable.
Heitmann et al. (41)	CC	Germany 2018	10^8^ PFU/ml	**+**	**-**	*Cx. molestus, Cx. pipiens* and *Cx. torrentium* infected but with no transmission detectable.
Hery, et al. (26)	CC	France 2019	10^7^ TCID_50_/ml	**-**	**-**	ZIKV strains tested were KU955592 (Senegal), KU647676 (Martinique) and KX694533 (Malaysia) at *Cx. quinquefasciatus*.
Hall-Mendelin et al. (27)	CC	Australia 2016	106.7 ± 0.2 TCID_50_/ml	**+**	**-**	A study performed with strain MR766. *Cx. sitiens* and *Cx. annulirostris* not infected. 7% of *Cx. quinquefasciatus* infected.
Duchemin et al. (19)	CC	Australia 2017	TCID_50_ 10^5.6^/ml	**-**	**-**	A study performed with *Cx. quinquefasciatus* and *Cx. annulirostris* and strain FSS13025.
Richard et al. (39)	CC	French Polynesia 2017	7 log_10_ TCID_50_/ml	**+**	**-**	No dissemination nor transmission for *Cx. quinquefasciatus*. A study performed with strain PF13/251013-18

*Ae*., *Aedes*; CA, conference abstract; CC, case-control; CH, cohort; *Cx*., *Culex*; DPI, days post-infection; ca., circa; Exp., experiment; FFU (fluorescence focus units); MC, mosquitoes collected from the field; blood meal titers expressed as PFU (plaque-forming unit) and PFUe (plaque-forming unit equivalents); NT, not tested; SG, Salivary glands; TCID_50_ (50% tissue culture infectious dose). Table prepared by the authors.

### Vector competence

Thirteen studies concluded that *Culex* spp. are not ZIKV competent vectors because they cannot be orally infected (14-26). These studies used a variety of ZIKV strains, including PRVABC59 from Puerto Rico (2015), FSS13025 from Cambodia (2010), MEX1-7 from Mexico City, Mexico (2015), DAKAR 41525 from Senegal (1985), PF13/251013-18 from French Polynesia (2013), BRPE243 from Pernambuco, Brazil (2015), SPH2015 from São Paulo, Brazil (2015), NC-2014-5132 from New Caledonia (2014), and strains with GenBank number KU955592 from Senegal, KU647676 from Martinique, and KX694533 from Malaysia. Five studies (from Sardinia (Italy) (15), Chiapas (Mexico) (16), San Juan (Puerto Rico) (21), and Singapore (25, 26)) analyzed field-caught mosquitoes, not ones from laboratory colonies. The blood meal titers used to feed the mosquitoes in colonies ranged from 1 × 10^4^ to 1 × 10^7^ focus-forming units per milliliter (FFU/ml), 1 × 10^7.3^ to 1 × 10^8.4^ plaque-forming units per milliliter (PFU/ml), and 1 × 10^5.6^ to 1 × 10^7.52^/ml 50% tissue culture infectious doses (TCID_50_). *Culex annulirostris* (Skuse) was identified as not susceptible to ZIKV infection in a single study (27), unlike *Cx. quinquefasciatus*, *Culex pipiens* (Linnaeus), and *Culex tarsalis* (Coquillett), each of which was identified as being infected in at least in one additional paper.

In another 16 studies, ZIKV infection was confirmed but without infected salivary glands or transmission (27-42). In a study conducted in Kédougou, Senegal (30), the ZIKV was identified in a single female *Culex perfuscus* (Edwards) mosquito. After this identification, the authors assumed a potential transmission capacity, but they did not perform any specific identification tests on the salivary glands. The infected virus strains in *Culex*. spp. were: H/PF from French Polynesia (2013), BE H 815744 from Brazil (2015), ZJ03 (GenBank No. 820899.2) from China (2016), GDZJ1685-2 from China (2016), PRVABC59 from Puerto Rico (2015), KFF993678 from Thailand (2013), RIO-U1 from Rio de Janeiro (2016), RIO-S1 from Rio de Janeiro (2016), PE243/2015 from the state of Pernambuco in Brazil (2015), Haiti 1225 from Haiti (2014), SPH2015 from São Paulo (2015), and strain MR766 from Uganda (1947). The infected mosquitoes were *Cx. quinquefasciatus*, *Cx. pipiens*, and *Cx. tarsalis*, with minimal blood meal titers of 5.7 log_10_ PFU/ml, 5.45 ± 0.38 log_10_ copies/ml and 7.0 log_10_ TCID_50_/ml.

Finally, seven studies identified ZIKV in the salivary glands of *Cx. restuans*, *Cx. quinquefasciatus*, *Cx. tarsalis*, and *Cx. coronator* (Dyar and Knab) (43-49). Detection of the virus genetic material occurred via real-time polymerase chain reaction (RT-PCR or RT-qPCR), with minimal blood meal titers of 4.0 log_10_ PFU/ml and 5.7 plaque-forming unit equivalents per milliliter (3.5 ± 0.1 log_10_ ZIKV PFUe/ml titer for freshly fed). These seven studies were performed in Hainan (province), China (11); Recife (city in the state of Pernambuco), Brazil (12, 43); Winnipeg/Beausejour (city and nearby town in the province of Manitoba), Canada (44); Guadalajara (city in the state of Jalisco), Mexico (45); Florida (state), United States of America (46); and Thailand (47). The study in Mexico (45) analyzed mosquitoes collected from the metropolitan area of Guadalajara but did not disclose the strains. This design was different from the studies in Brazil, Canada, China, Thailand, and the United States, which used mosquitoes from existing laboratory colonies. The Brazilian paper (43) also analyzed field-caught mosquitoes from Recife, Pernambuco, Brazil.

## DISCUSSION

In our work, we found 36 relevant studies. This was 18 more works than the already identified articles in five reviews in the literature that examined the capacity of *Culex* spp. to transmit ZIKV (3, 4, 8, 9, 48). *Cx. quinquefasciatus* was identified in 6 articles, while *Cx. restuans*, *Cx. tarsalis*, and *Cx. coronator* were identified as possibly competent in 1 study each.

As stated before, researchers from the Ageu Magalhães Institute have detected ZIKV in salivary glands of *Cx. quinquefasciatus* at 7 and 15 days after feeding, and confirmed an infection rate of 100% and 67%, respectively (12). Additionally, one Chinese paper (11) was the first to demonstrate the presence of ZIKV (Strain SZ01) in the salivary glands of *Cx. Quinquefasciatus;* it was also the only study to show the transmission capacity to another species. This work reported a transmission rate of 89% to mice; these animals had viral RNA in their brain at 10 days post-engorgement, with a titer of 7.85 RNA log_10_ copies/ml. On the other hand, this study did not provide experiments with the same methodology with *Ae. aegypti* as a positive control.

One study demonstrated the presence of ZIKV in the salivary glands of *Cx. quinquefasciatus* mosquitoes in concentrations similar (P > 0.05) to those found in *Ae. aegypti*, both fed with 1 × 10^6^ PFU/ml (log_10_) blood meal titers (43). Even with a minimum blood meal titer of 1 × 10^4^ PFU/ml (log_10_), *Cx. quinquefasciatus* salivary glands were infected. These findings indicate that *Cx. quinquefasciatus* can produce virus in the salivary gland even when fed with low titers of viral particles. This ability better mimics what occurs in nature, where mean human viremia is lower than 2.5 log_10_ PFU/ml (49). Likewise, the study in the Brazilian state of Pernambuco (43) investigated wild-caught mosquitoes from the city of Recife. They observed no traces of recent feeding and used electron microscopy to detect ZIKV in the salivary glands. That paper did not perform experiments to analyze ZIKV transmission from *Cx. quinquefasciatus* to another species.

Dibernardo et al. (44) detected ZIKV in 3 of 58 (5%) of salivary glands of *Cx. restuans* just after intrathoracic inoculation. *Cx. tarsalis* was refractory using the same methodology. The authors believe that *Cx. restuans* could transmit by bite but also suggested the presence of salivary and midgut barriers for *Cx. restuans* and *Cx. tarsalis*. They concluded that *Cx. restuans* is not a competent ZIKV vector due to its feeding behavior.

Elizondo-Quiroga et al. (45) detected ZIKV in wild-caught *Cx. tarsalis*, *Cx. coronator*, and *Cx. quinquefasciatus*. However, this study was not conclusive regarding the ZIKV infection titers in the saliva of the collected mosquitoes because the mosquitoes that presented the lowest maximum titer of infection were *Ae. aegypti*, the primary ZIKV vector. Unfortunately, for none of those mosquitoes did the authors indicate whether the mosquitoes had any trace of recent feeding or which ZIKV strains were involved. Such deficiencies impair a more detailed assessment of the competence of these *Culex* spp.

RNA analysis was performed in saliva from *Cx. quinquefasciatus* eluted from filter paper at 14 days postinfection (DPI) in a first experiment and then at 16 DPI in a second experiment (46). The ZIKV titers were 5.6 ± 4.5 log_10_ ZIKV PFUe/ml and 5.02 log_10_ ZIKV PFUe/ml, respectively. This same research group (46) neither investigated transmission to another species nor applied the same methodology for *Ae. aegypti* (as a positive control). The lack of positive controls diminishes the reliability of the results. On the other hand, this paper intriguingly showed that viruses isolated from *Culex* saliva can form plaques in Vero cells. These data prove that biologically active virus can be obtained from the saliva of those mosquitoes.

A Thai study investigated vertical transmission of ZIKV to larvae in *Ae. aegypti*, *Ae. albopictus, and Cx. quinquefasciatus*, with positive results for all the species (47). The ZIKV strain virus/*H. sapiens*-tc/THA/2014/SV0127-14 infected the salivary glands of these mosquitoes fed with 1.7x10^5^ FFU/ml blood meal titers. These Thai investigators (47) did not demonstrate any kind of transmission to other species but applied the same methodology for *Ae. aegypti* as a positive control.

Six studies that examined mosquito colonies reported that only when infection rates (IRs) from bodies were over 50% was salivary gland infection noted (11, 12, 43, 44, 46, 47). In addition, we found 13 other studies with IRs under 50% without any detected transmission capacity (28-32, 34-41). One article, with a range of 15% to 70% of mosquitoes with intrathoracic inoculation, demonstrated virus-positive bodies but no detectable viral RNA or infectious virus in saliva (38).

In two studies (43, 46), the infection of salivary glands declined over time, findings that are different from the Chinese study mentioned earlier (11). Thus, there are inconsistent results with *Cx. quinquefasciatus*, with a marked decrease from day 8 to day 12 postexposure, but an apparent increase again at days 16 and 18 postexposure. Another aspect in these three articles is that they all used different strains: the Chinese study (11) used SZ01, from an infected patient who had returned from Samoa to China; the Brazilian paper (43) used BRPE243/2015, from Pernambuco; and the United States paper (46) used PRCABC59, from Puerto Rico (2015). Another study about vector competence of *Aedes* mosquitoes argued that in *Ae. albopictus* and *Ae. aegypti*, ZIKV transmission can be relatively dependent on the virus strain (49). Thus, it is important to note that diverse strains, especially strains isolated from patients, can present distinct behaviors. This phenomenon may represent one factor responsible for the varied results found in the literature.

With regards to blood meal titers, only one work (43), which used 4.0 log_10_ ZIKV PFU/ml, reached an IR of 36% at 7 DPI and 10.53% at 15 DPI. The majority of studies that reached some IR used blood meal titers greater than 5.0 log_10_ ZIKV PFU/ml, 5.0 log_10_ ZIKV RNA copies/ml, or 1 × 10^6.7^ TCID_50_/ml. The results were refractory at lower blood meal concentrations. As described above, 13 studies did not detect the infection capacity, dissemination, or transmission of any *Culex* spp., even with higher blood meal titers. Some authors (28, 38, 44) suggested that the random inability to transmit in *Culex* mosquitoes may be linked to a gut barrier of some *Culex* spp., a place where viral particles attack and initiate penetration and replication. However, Amraoui et al. (28) did not demonstrate that inoculation of viral particles into the hemocoel tissue of *Cx*. *quinquefasciatus* favored viral ZIKV dissemination or transmission. A study from the United States (46) warned about the existence of specific populations with regard to variability in transmission competence. The authors concluded that some *Cx. quinquefasciatus* populations may be capable of salivating ZIKV under environmental and other unknown conditions. This statement is noteworthy. It cannot be ignored that some mosquito populations may be more prone to ZIKV infection and dissemination. All these aspects definitely merit further studies that could reveal new intervention approaches.

Another particular condition revealed by Ciota et al. (49) in a study on the vector competence of *Aedes* mosquitoes was that there were significant differences in the proportion of infected mosquitoes with equivalent ZIKV titers but two different types of meals. Fresh blood meals resulted in a significantly higher IR than did stocked meals frozen and stored at -80°C and then thawed before preparation (P < 0.0001). Thus, considering the documents of Table 1 that indicated the transmission capacity, the Brazilian work in Pernambuco (43) stored the viral stocks at -80°C and subsequently thawed them to prepare the blood meal. However, another paper (11) was unclear about storage conditions and used a stock of virus that had been passaged twice in C6/36 cells prior to the infectious feed. This method suggests the use of a recently prepared blood meal.

Roundy et al. (50) noted that the criterion (iii) proposed by Barnett (51) for incrimination of an arthropod vector (repeated demonstration of natural infection of the vector) has only been fulfilled for *Ae. aegypti* and *Ae. albopictus*. Furthermore, as can be seen in our review, only one study (11) demonstrated ZIKV transmission to other species (criteria (iv) for incrimination of an arthropod (51)). However that study used a ZIKV strain not tested in any other work found for this review, and it did not include *Ae. aegypti* positive control tests**. Thus, the defining evidence for *Culex* spp. as a ZIKV vector is still lacking.

Some authors (32, 38, 40) agree that the focus on prevention of ZIKV disease should remain on population control of the genus *Aedes*. Indeed, the probability of a *Culex* mosquito biting two humans in a sequence and transmitting the Zika virus is small, according to its preference for feeding on avian hosts (52). Nevertheless, *Culex* spp. are widespread in urban centers and also feed on human blood. Considering the results of studies with collected mosquitoes, when the *Culex* spp. was infected, the *Aedes* species was infected (30, 42, 43, 45). Only one study (15) presented data that showed neither *Culex* spp. nor *Aedes* mosquitoes infected with ZIKV. Conversely, in four papers (16, 24, 25, 26), there was no *Culex* mosquito infected while the official vector *Ae. aegypti* was infected. Two articles (25, 26) used the same collection of mosquitoes from the field with different methodologies to investigate viral ZIKV RNA. All these results with field-caught mosquitoes showed that *Culex* spp. and *Ae. aegypti* may use identical hosts (but not always). In fact, in a study with mosquitoes from field collections in Thailand (53), in *Cx. quinquefasciatus* there were mixed blood meals, with 7.84% from humans or monkeys, 47.06% from dogs, and 33.33% from others hosts. Comparatively, in *Ae. aegypti*, there were also several blood meals: 70.0% from humans plus monkeys or 13.33% only from monkeys and 10.0% from other kinds of hosts. These data demonstrate that variations in favorite hosts from place to place influence the infection rates of *Culex* spp. and *Aedes* mosquitoes. Additionally, as argued by Kauffman et al. (8), especially regarding the work from Pernambuco, Brazil (43), *Cx. quinquefasciatus* may serve as a secondary vector in places with abundant ZIKV infection in humans.

Additional experimental studies that use identical strains, experimental conditions, methodologies, with positive controls in *Ae. aegypti* and/or *Ae. albopictus*, and that utilize tests to prove the possibility of ZIKV transmission to other species from *Culex* spp., could be very decisive for discarding or confirming the contribution of these mosquitoes as a competent or incompetent ZIKV vector. According to the studies we have investigated, we feel that, besides well-implemented sanitization, the main strategies for the prevention and control of ZIKV should remain on the genus *Aedes*.

## Conclusions

This work demonstrated the accumulation of evidence to prove the capacity of the ZIKV to infect *Culex* spp. However, only 7 studies out of the 36 identified for this review demonstrated the infection of *Cx. restuans*, *Cx. quinquefasciatus*, *Cx. tarsalis*, and *Cx. coronator* salivary glands. Furthermore, only 1 study showed the capacity of transmission to mice. Considering the records found here, *Cx. quinquefasciatus* remains the most widely studied species with confirmed salivary glands infected by ZIKV.

Additionally, only Asian or American ZIKV strains were able to infect the salivary glands of *Culex* mosquitoes: THA/2014/SV0127-14, SZ01, BRPE243, and PRVABC59. The MR766 and DAK AR 41525 African strains were unable to infect *Culex* spp. Further experimental studies that utilize the same strains, experimental conditions, use a positive *Aedes* control, and test ZIKV transmission to other species via *Culex* spp. are still needed to confirm the contribution of the *Culex* mosquitoes in ZIKV transmission. We believe that strategies for ZIKV control should stay focused on the genus *Aedes,* but responsible authorities should continue to monitor *Culex* spp. mosquitoes, especially regarding their ability to transmit ZIKV to other species. This surveillance should not be limited to determining whether their salivary glands are infected.

### Author contributions.

All the authors (SGVR, EGR, and WCS) conceived the original idea, collected the data, analyzed the data and interpreted the results, and wrote the paper. All the authors reviewed and approved the final version.

### Disclaimer.

Authors hold sole responsibility for the views expressed in the manuscript, which may not necessarily reflect the opinion or policy of the *RPSP/PAJPH* and/or PAHO.
